# Prevalence and Correlates of Depression Among Pregnant Women at King Abdulaziz Medical City: A Tertiary Hospital in Riyadh, Saudi Arabia

**DOI:** 10.7759/cureus.56180

**Published:** 2024-03-14

**Authors:** Mouath A Alturaymi, Awatef Alsupiany, Omar F Almadhi, Khalid M Alduraibi, Yazeed S Alaqeel, Mohammed Alsubayyil, Majed Bin dayel, Saad Binghanim, Bader Aboshaiqah, Fahad Allohidan

**Affiliations:** 1 Medicine, King Saud Bin Abdulaziz University for Health Sciences, Riyadh, SAU; 2 Mental Health, King Abdulaziz Medical City, Riyadh, SAU

**Keywords:** mental health, saudi arabia, prevalence, pregnancy, depression

## Abstract

Background: Women have a higher likelihood of experiencing depression during pregnancy due to the significant physiological and mental changes that occur during this critical period. The frequency of antenatal depression varies globally according to socioeconomic, healthcare, and cultural influences. The objective of this study is to investigate the prevalence of depression among pregnant women in Riyadh, Saudi Arabia.

Materials and methods: Women who were pregnant and who had undergone screening for depression were included in this cohort study that was conducted at King Abdulaziz Medical City (KAMC). Using a non-probability convenience sample technique, data was obtained from the hospital record system. Statistical significance was determined using Fisher's exact test and student's t-test, which analyzed demographic, clinical, demographic, and obstetric information. Significance was determined by a P-value that was lower than 0.05.

Results: Among 367 people surveyed, the prevalence of depression was 2.5%. This is much lower than rates seen both globally and in Saudi Arabia. The majority were married and non-smokers with a high post-delivery BMI. A significant association was observed between depression and previous psychiatric diagnoses, while no significant relationships were found with gestational age, complications, chronic illnesses, or referral sources.

Conclusion: The research shows that the rate of perinatal depression among women in Riyadh is much lower than the average, highlighting the critical role of cultural factors and the need for validated, culturally sensitive screening tools. Recommendations for future research include longitudinal studies and the development of culturally tailored interventions to enhance the detection and management of depression during pregnancy, integrating mental health care into routine antenatal services.

## Introduction

Pregnancy is a complex and highly emotional process in the lives of many women. There are many alterations in physiological and emotional well-being that occur during this period. Mothers could start experiencing these changes from the beginning of pregnancy until the postpartum period [1,2]. However, maternal stressors during pregnancy are associated with many adverse effects, including preeclampsia, preterm delivery, low neonatal birth weight, and neonatal morbidity [3].

Depression is a mood disorder that causes a persistent feeling of sadness and loss of interest. It has a wide range of symptoms that might influence physical, cognitive, emotional, and social activities. Depression is one of the most common mental health disorders globally, affecting approximately 280 million patients, which is approximately 3.76%, and 5.7% of them are above 60 years of age. It is also more associated with women than with men [4]. Furthermore, depression rates are more notable during the second and third trimesters [5,6]. It is well documented that depression is the most common psychological issue that has an impact on women worldwide during the perinatal period [7-9].

On the other hand, locally, a study was done in December 2016 in Saudi Arabia by the King Salman Center for Disability Research with a sample size of 4,004 males and females across the country, and their ages are between 15 and 65 by using the Saudi version of the Composite International Diagnostic interview questionnaire. Surprisingly, depression was the third most common mental health condition in the Kingdom of Saudi Arabia (KSA) across a lifetime affecting about 6% of the population [10].

Additionally, depression can be one of the pregnancy complications [11]. Regarding the prevalence of antenatal depression, globally, a study published in February 2020 that included 10 reviews (306 studies with 877,246 participants) on antenatal depression prevalence and six reviews (39 studies with 75,451 participants) conducted to identify the effect of antenatal depression on preterm and low birth weight. Furthermore, the results were that the prevalence of depression among pregnant ranges from 15% to 65% [12]. Furthermore, regarding the prevalence of depression among pregnant women, in a study done in Ethiopia, in 393 pregnant patients, 24.94% of them had depression [13]. Moreover, a study conducted in the USA measuring the prevalence of depression among pregnant and non-pregnant women found that major depression is less in pregnant than in non-pregnant, but the presence of minor depression in pregnant women is more [14]. Locally, three studies were conducted in KSA about the prevalence of antenatal depression. The result of the study is that the range of the prevalence among them is between 26.8% and 37.5% [2,15,16].

Perinatal depression is connected with high morbidity and mortality; therefore, determining the risk factor for it is a good tool to detect depressed pregnant women, or those who are prone to perinatal depression [17]. Thus, a systematic review study demonstrates that the risk factors of perinatal depression can be low educational level, poor economic status of families, history of mental illness, domestic violence, perinatal smoking or drinking, and multiparity [17]. Also, another systematic review study assessed proved that the risk factors were associated with previous or recurrent exposure to any type of abuse and violence (six reviews and 73 studies), lack of social and/or partner support (four reviews and 47 studies), and personal or family history of any common mental disorder (three reviews and 34 studies) [12]. Also, depression in pregnancy is linked to many complications such as low birth weight, preterm birth, growth problems, and cognitive complications [11].

In addition, regarding the management of antenatal depression, between 1% and 13% of pregnant women get medical management, as indicated by antidepressant prescriptions for their depression [18,19]. Cessation of any type of depression management is very common during pregnancy. According to a study, the rate of antidepressant prescription is around 70% before pregnancy of women with depression. This rate goes down to approximately 27% during pregnancy [5,19]. Finally, in this study, we aim to measure the prevalence of depression in pregnant women who were screened in King Abdulaziz Medical City (KAMC).

## Materials and methods

The study was conducted in King Abdulaziz Medical City (KAMC) which is located in Riyadh, Saudi Arabia. In the National Guard Hospital, we have recently established a comprehensive screening program for depression as part of antenatal care using the Edinburg scale to provide the appropriate care. Edinburgh Postnatal Depression Scale (EPDS) is a valid self-reported questionnaire to detect depression in antenatal period of pregnancy [20]. Five women with EPDS of 10 or higher may have depression; the higher the score, the higher the severity of depression. A non-probability convenience sampling technique was used to collect the data.

An electronic data extraction was done retrospectively from the National Guard Hospital patient record system (best care) in a random fashion. We included all patients who came to either family medicine or obstetric clinics for antenatal care, which is screened by the Edinburgh score for depression. Demographic data like age, marital state, education level, employment, and living situation was examined, which was already recorded in the pregnancy diary already filled by a primary physician for each pregnant lady in the National Guard Health Services (NGHS) as part of antenatal care. This study had inclusion criteria which included all pregnant women who were screened in King Abdulaziz Medical City (KAMC). On the other hand, all pregnant women who were not screened for depression and who were from other health facilities were excluded.

The study included all patients who fulfilled the inclusion/exclusion criteria stated in 11.2 from February 2022 to December 2022. Then, Raosoft was used to determine the sample size at a margin of error of 5%. The confidence interval was set at 95%. Population size is approximately 4.5 million, and the response distribution was set at 50%.

The confidentiality and privacy of patient’s data were maintained by making sure that patient-specific data such as medical record number (MRN) or patient name was not used; instead, each patient was given a serial number. No informed consent was taken because it was a chart review, and no patient contact was there. Also, any data that is not related to the research topic was not used and remained confidential. Data was collected after King Abdullah International Medical Research Center (KAIMRC) approval.

Data was entered and analyzed using the Statistical Package for the Social Sciences (SPSS) software version 23 (IBM SPSS Statistics 26). Data cleaning and assigning codes were done before the analysis. Descriptive statistics were presented as frequency and percentages for the categorical variables and as mean ± standard deviation for the numerical variables. Student’s t-test and Fisher's exact test were used to compare means and proportions, respectively. A P-value less than 0.05 was considered statistically significant.

## Results

A total of 367 pregnant women were included in the current study. Most of the participants were in the age group of 20 to 30 years (44.7%) and the age group of 31 to 40 years (43.9%). Regarding their marital status, the vast majority of them were married (99.5%) during the period of the study. Also, the majority of the participants were non-smokers (97.5%). The average post-delivery body mass index of the participating women was 30.5 ± 6.0 ranging from 17.4 to 48.4 kg/m^2^. About 86.1% of the participants had children, while 10.9% were not, and the average number of children was 3 ± 2. The mean number of gravidity was found to be 3.5 ± 2.4 and 2.7 ± 1.8 for parity. Abortion was reported in 37.9% of the participants, and the average number of abortions was 1.7 ± 1.4. The mean gestational age of the participants was 34.1 ± 8.8 (Table 1).

**Table 1:** Characteristics of the Study Participants (n=367)

Continuous variables	Mean ± SD
Post-delivery body mass index (kg/m^2^)	30.5 ± 6.0
No. of children	3 ± 2
Abortion	1.7 ± 1.4
Gestational age (in weeks)	34.1 ± 8.8
Abortion	1.7 ± 1.4
No. of parity	2.7 ± 1.8
Categorical variables	n (%)
Age (in years)	
Less than 20	4 (1.1)
20-30	164 (44.7)
31-40	161 (43.9)
41-50	38 (10.4)
Marital status	
Married	365 (99.5)
Divorced	1 (0.3)
Widowed	1 (0.3)
Smoking	
Yes	2 (0.5)
No	358 (97.5)
Second hand	7 (1.9)
Having children	
Yes	316 (86.1)
No	40 (10.9)
Missing data	11 (3)
Parity	
No	44 (12)
Yes	320 (87.2)
Missing data	3 (0.8)
Abortion	
No	222 (60.5)
Yes	139 (37.9)
Missing data	(1.6)

**Figure 1:**
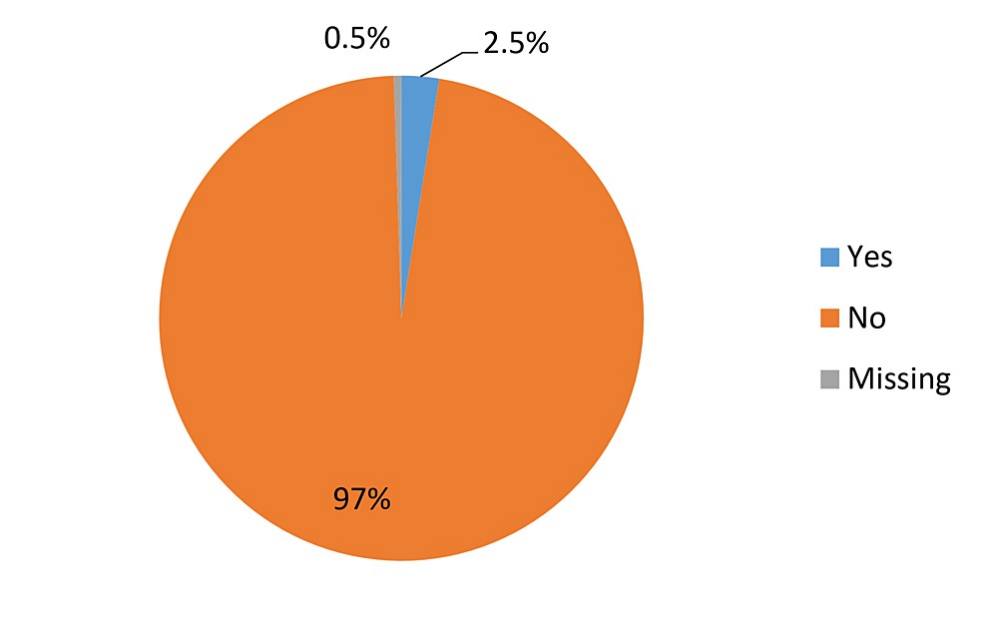
Prevalence of Depression Among Pregnant Women

**Figure 2:**
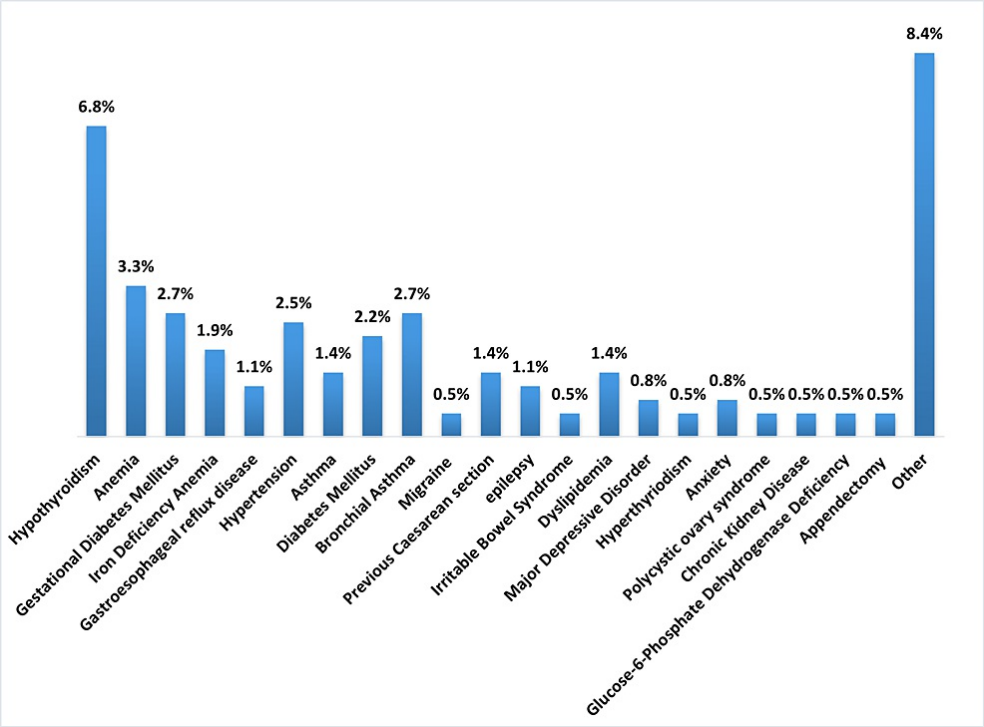
Medical Disorders and Chronic Illnesses

Regarding the prevalence of depression among pregnant women, it was found that nine (2.5%) pregnant women had depression (Figure 1). Concerning pregnancy complications, most of the participants (65.1%) had no complications, and among participants who experienced complications, the most reported one was gestational diabetes mellitus (10.6%) followed by C-section (6%). The vast majority of the participants had no history of psychiatric diagnosis (95.6%), while 1.4% had anxiety, and the same percentage had major depressive disorder (MDD). About suicidality, one (0.3%) had ideation and one (0.3%) had previous attempts. About 33.5% of the participants had medical disorders and chronic illnesses, and the most reported chronic illness was hypothyroidism (6.8%) followed by anemia (3.3%), gestational diabetes mellitus (GDM) and bronchial asthma (BA) (2.7% for each), hypertension (2.5%), and diabetes mellitus (2.2%) (Figure 2). Nearly, all the participants had no family history of perinatal depression (99.7%). There was no referral source for about a third of the participants (33.2%), and for 29.7%, the referral source was obstetrics and gynecology physician, and for 28.6%, the referral source was family medicine. Only 4.6% of the participants had been referred to psychiatry, while 94.8% were not. The most reported reason for referral to psychiatry was clinical assessment (4.9%) (Table 2).

**Table 2:** Pregnancy Complications and Psychiatric History

		n (%)
Complications	None	239 (65.1)
	Cesarean section	22 (6)
	Gestational diabetes mellitus	39 (10.6)
	Postpartum hemorrhage	7 (1.9)
	Pre-eclampsia	6 (1.6)
	Oligohydramnios	5 (1.4)
	Fetal distress	3 (0.8)
	Preterm rupture of membrane	3 (0.8)
	History of breech presentation	3 (0.8)
	Congenital anomaly	2 (0.5)
	Gestational hypertension	2 (0.5)
	Thrombocytopenia	2 (0.5)
	Other	19 (5.2)
Previous psychiatric diagnosis	None	351 (95.6)
	Anxiety	5 (1.4)
	Major depressive disorder	6 (1.7)
	Postpartum depression	2 (0.5)
	Generalized anxiety disorder	1 (0.3)
	Panic disorder	1 (0.3)
	Physical abuse	1 (0.3)
	Psychotic depression	1 (0.3)
Suicidality	No	358 (97.5)
	Ideation	1 (0.3)
	Previous attempt	1 (0.3)
	Missing data	7 (1.9)
Having medical disorders and chronic illnesses	Yes	123 (33.5)
	No	242 (65.9)
	Missing data	2 (0.5)
Family history of perinatal depression	No	366 (99.7)
	Missing data	1 (0.3)
Referral source	None	122 (33.2)
	Family medicine	105 (28.6)
	Obstetrics and gynecology physician	109 (29.7)
	Other specialties	27 (7.4)
	Emergency medicine	2 (0.5)
	Genetic	1 (0.3)
	Missing data	1 (0.3)
Referral to psychiatry	Yes	17 (4.6)
	No	348 (94.8)
	Missing data	2 (0.5)

To investigate the factors associated with depression among pregnant women, we conducted Fisher's exact test and student's t-test to find the statistically significant factors. We found that there is a significant association between previous psychiatric diagnosis and depression during pregnancy (P < 0.001); a higher prevalence of depression was observed among women having previous psychiatric diagnosis compared to those without psychiatric diagnosis (26.7% vs. 1.4%). Other factors including gestational age, complications, chronic illnesses, and referral source did not show any significant relationship with depression during pregnancy (Table 3). Based on statistical data displayed in Table 3, it is evident that obstetric and gynecological physicians accurately diagnosed depression among women during pregnancy in six cases (5.5%). However, family medicine physicians managed to diagnose only two cases (1.9%).

**Table 3:** Factors Associated With Depression Among Pregnant Women ER: emergency department, Ob gyne: obstetrics and gynecology.

Variable	Diagnosis with depression during pregnancy		P-value
	Yes	No	
Gestational age (in weeks): mean ± SD	37.6 ± 3.68	34.0 ± 8.91	0.232
	n (%)	n (%)	
Complications			
Yes	4 (4.1)	93 (95.9)	0.456
No	5 (2.1)	233 (97.9)	
Having chronic illnesses			
Yes	2 (1.6)	120 (98.4)	0.514
No	7 (2.9)	234 (97.1)	
Previous psychiatric diagnosis			
Yes	4 (26.7)	11 (73.3)	<0.001*
No	5 (1.4)	345 (98.6)	
Referral source			
None	0 (0)	121 (100)	0.120
Family medicine	2 (1.9)	103 (98.1)	
Ob gyne physician	6 (5.5)	103 (94.5)	
3	1 (3.7)	26 (96.3)	
ER	0 (0)	2 (100)	
Genetic	0 (0)	(100)	

## Discussion

This study used the Edinburgh Postnatal Depression Scale (EPDS) to assess the prevalence of depression among pregnant women. Most of the participants were in the 20-40 age range, reflecting a typical childbearing age, and nearly, all were married, which aligns with cultural norms in Saudi Arabia. The high average postpartum BMI indicates an upward trend toward overweight and obesity within the cohort and conforms to the overall picture of global changes in maternal health and may potentially contribute to the development of a depressive state [21]. The reported figures of children, gravidity, and parity all offer an important background for understanding the participants’ reproductive history, which may be of relevance to depression. The number of abortions, which has become a defining characteristic of this cohort, makes it important to delve into the possible psychological impacts of this side of social phenomenon [22].

Our results showed a prevalence of 2.5%, in the sample, that is much lower than what was reported previously both worldwide and in Egypt. For instance, a study published during the COVID-19 pandemic reported that 44.6% of the pregnant population suffers from depression [23]. Several studies have shown an increased prevalence of depression between 35% and 37.2%, which is higher than our study's lower prevalence rate [24]. Depressive symptoms were present in 44.2% of pregnant women in Saudi Arabia, according to one research [25]. Among pregnant women in Egyptian research, 63% reported experiencing depressive symptoms [26]. Additionally, a reduced prevalence rate of depression has been shown in several studies. For example, a New Zealand study found a lower prevalence of depression to be 22% [27], and in a Spanish study, the prevalence rate of depression based on the trimester was 21.5%-22% [28]. However, this prevalence is still higher than our findings.

According to Fisher et al. (2012), pregnant women with depression experience it just as often, if not more often, in high-income nations as in low-income ones. Different prevalence rates may arise due to differences in research context, sampling demographic, or measurement and evaluation methods [29]. Several studies have identified variables that are either linked to or increase the occurrence of prenatal depression. An increased risk of severe depression during pregnancy is 22.4 times higher in women with a history of depression, making preexisting depression a substantial risk factor for major depression during pregnancy [30]. This can, however, be compared to our findings because we found patients with previous psychiatric disorders have a higher risk to develop depression during pregnancy. There are several factors that may contribute to a reduced prevalence reported in our study, including differences in screening tools, study populations, and cultural perceptions of mental health. The EPDS is a well-validated screening instrument to detect depression in pregnant women, yet its sensitivity and specificity may change according to the cultural and linguistic setting. In addition, social discrediting of mental disorders in Saudi Arabia may result in the underreporting of depressive symptoms among pregnant women while contributing to the low prevalence reported [31].

In our findings, previous psychiatric diagnoses were also associated significantly with depressive episodes during pregnancy with a higher prevalence of depression recorded in the women who had psychiatric diagnoses. This result concurs with what is known in the literature as a notable risk factor for the common occurrence of antepartum depression is a long history of mental illness [2,28]. It emphasizes the need for detailed psychiatric assessments in pregnant women with any history of mental health problems to promote early interventions and support measures.

In terms of the lack of considerable statistical associations between depression, other variables such as gestational age, pregnancy complications, chronic illnesses, and referral sources in our study differ from what is reported in some prior studies. For instance, as shown in a review by Bennett et al. (2004), poor social support, domestic violence, and adverse life events were good predictors of perinatal depression [5]. Our results provide evidence of the possible difference in effect across the populations and stress the necessity of specific studies on antenatal depression that was seen to be quite complex.

A critical limitation of this study lies in the absence of a comprehensive array of clinical screening tools, which restricts our ability to detect varying degrees and types of depression. The singular screening tool utilized in this study, while beneficial for initial assessments, may not reflect the experiences of individuals with depression. Additionally, the prevalent cultural stigma surrounding mental illnesses likely aggravates reporting biases, preventing individuals from reporting their symptoms. This combination of methodological limitations and societal barriers suggests that the observed low prevalence rates of depression could significantly underestimate the actual figures. Moreover, a lack of longitudinal data limits the analysis of the trajectory of depression or the effect that antenatal depression may have on outcomes postpartum, indicating that more research using a variety of methodologies and longitudinal approaches is needed to fully understand and approach the issue of antenatal depression in this population.

## Conclusions

Our study highlights a lower prevalence of depression among pregnant women in Riyadh and emphasizes the significance of cultural considerations in mental health screening. The association between depression and prior psychiatric history suggests the need for targeted screening strategies. Recommendations include adopting longitudinal research designs to better understand depression's trajectory and implementing culturally tailored interventions to improve detection and management. This work underlines the imperative for integrating mental health care into antenatal services, addressing both the stigma and the specific needs of pregnant women in Saudi Arabia.
